# Application of Rapeseed Meal Protein Isolate as a Supplement to Texture-Modified Food for the Elderly

**DOI:** 10.3390/foods12061326

**Published:** 2023-03-20

**Authors:** Gabriella Di Lena, Ann-Kristin Schwarze, Massimo Lucarini, Paolo Gabrielli, Altero Aguzzi, Roberto Caproni, Irene Casini, Stefano Ferrari Nicoli, Darleen Genuttis, Petra Ondrejíčková, Mahmoud Hamzaoui, Camille Malterre, Valentína Kafková, Alexandru Rusu

**Affiliations:** 1 CREA Research Centre for Food and Nutrition, Via Ardeatina 546, 00178 Rome, Italy; massimo.lucarini@crea.gov.it (M.L.); paolo.gabrielli@crea.gov.it (P.G.); altero.aguzzi@crea.gov.it (A.A.); roberto.caproni@crea.gov.it (R.C.); irene.casini@crea.gov.it (I.C.); stefano.nicoli@crea.gov.it (S.F.N.); 2 Biozoon GmbH, Nansenstraße 8, 27572 Bremerhaven, Germany; schwarze@biozoon.de (A.-K.S.); genuttis@biozoon.de (D.G.); rusu@biozoon.de (A.R.); 3 ENVIRAL A.S., Trnavská Cesta, 920 41 Leopoldov, Slovakia; ondrejickova@enviengroup.eu; 4 Celabor, Avenue du Parc 38, 4650 Herve, Belgium; mahmoud.hamzaoui@celabor.be (M.H.); camille.malterre@celabor.be (C.M.); 5 Centrum Výskumu a Vývoja, s. r.o. (Centre for Research and Development), Trnavská Cesta 1033/7, 920 41 Leopoldov, Slovakia; kafkova@enviengroup.eu

**Keywords:** agri-food by-products, rapeseed meal, sustainable protein, food for elderly, texture-modified food, nutritional value, sensory quality

## Abstract

Rapeseed meal (RSM), a by-product of rapeseed oil extraction, is currently used for low-value purposes. With a biorefinery approach, rapeseed proteins may be extracted and recovered for high-end uses to fully exploit their nutritional and functional properties. This study reports the application of RSM protein isolate, the main output of a biorefining process aimed at recovering high-value molecules from rapeseed meal, as a supplement to texture-modified (TM) food designed for elderly people with mastication and dysphagia problems. The compositional (macronutrients by Official Methods of Analyses, and mineral and trace element profiles using Inductively Coupled Plasma Optical Emission Spectrometry ICP-OES), nutritional and sensory evaluations of TM chicken breast, carrots and bread formulated without and with RSM protein supplementation (5% *w/w*) are hereby reported. The results show that the texture modification of food combined with rapeseed protein isolate supplementation has a positive impact on the nutritional and sensory profile of food, meeting the special requirements of seniors. TM chicken breast and bread supplemented with RSM protein isolate showed unaltered or even improved sensory properties and a higher nutrient density, with particular regard to proteins (+20–40%) and minerals (+10–16%). Supplemented TM carrots, in spite of the high nutrient density, showed a limited acceptability, due to poor sensory properties that could be overcome with an adjustment to the formulation. This study highlights the potentialities of RSM as a sustainable novel protein source in the food sector. The application of RSM protein proposed here is in line with the major current challenges of food systems such as the responsible management of natural resources, the valorization of agri-food by-products, and healthy nutrition with focus on elderly people.

## 1. Introduction

Protein availability in the future will not be sufficient to meet the increased demands of a growing world population, expected to reach 10 billion people by 2050 [1]. Therefore, the exploitation of new sustainable sources of proteins has become an emergent issue in the agri-food and human nutrition sectors [2].

With the need for sustainable and nutritionally valid protein sources, there is increasing interest in plant-based proteins, characterized by a lower environmental impact in terms of greenhouse gas emissions, water requirements, and land use compared to animal proteins [3].

The adoption of plant-rich dietary patterns not only benefits the planet by lowering the environmental impact of food production, but has also a positive impact on public health by reducing the risk of chronic diseases associated with animal-based dietary patterns [4,5,6,7,8]. For the total or partial replacement of animal proteins in the diet, plant protein choices with good performance, both in techno-functional and nutritional terms, are the preferred ones. In some cases, traditional and novel technologies (i.e., fermentation, enzyme technology, and nanotechnology) may aid in improving the physico-chemical, sensory, and nutritional properties of plant food [9,10,11].

Agri-food by-products represent a highly sustainable source of nutrients and bioactive compounds that, with a circular economy approach, may be upgraded to fully exploit their inherent value. Oilseed processing by-products are a class of agrifood by-products attracting growing interest because of their high content in proteins with excellent nutritive properties and for the opportunities they offer to develop healthy foods or food supplements [12].

Rapeseed meal (RSM), a by-product of rapeseed oil extraction connected to the agrifood and biofuel sectors, is produced in large quantities worldwide (40 million tons in 2020) and in the EU (12.5 million tons in 2021/2022 and 13.0 million tons expected in 2022/2023) [13]. Future projections indicate a constant growth of rapeseed oil production, with proportional increments of this by-product, currently used as animal feed and for other low-value purposes [13,14].

In a previous study, we provided a chemical characterization of RSM produced by an oilseed-producing factory serving a biofuel plant in view of its exploitation and valorization. The study highlighted the potentialities of this rapeseed oil co-product to establish new cross-sector interconnections between the biofuel and food value chains [15].

RSM is an interesting source of sustainable proteins with a complete amino acid profile and peculiar functional properties. It is recognized as a promising ingredient for food product formulation when protein fortification, modulation of the rheological properties, or replacement of animal proteins are desired [16,17,18]. Other advantages of using rapeseed proteins in food formulations are connected with their techno-functional properties, i.e., water-holding, gelling, emulsioning, and foaming properties, reported to be comparable or even higher than those of conventional proteins such as casein, soybean, or egg proteins [19,20].

With a biorefinery approach, RSM could be valorized as a sustainable protein source for high-value applications, opening perspectives to the full and sustainable exploitation of this residual biomass. One of the challenges connected with the utilization of rapeseed proteins is their extraction in an economically and environmentally sustainable way and also in mild conditions so as to retain as much the native properties of proteins as possible.

Studies reporting the uses of rapeseed proteins in food formulation move from the assumption that they are a suitable and promising ingredient for vegetarians, vegans, and consumers concerned with a reduction in the environmental impact of their diet. Several studies have been carried out in recent years on the applications of rapeseed/canola proteins in food products as a partial or total replacement of animal proteins. Depending on the food application tested, rapeseed proteins have been proposed as a thickener ingredient or as an emulsifier, binder, foaming, or gelling agent able to modify texture or simply to fortify the protein content of a product. The range of possible food applications for rapeseed/canola proteins include bakery and dairy products, meat, confectionery and beverages, as well as dressings, sauces, snacks or flavorings [21,22,23,24,25,26,27].

With the increased life expectancy registered worldwide, it is assumed that, by 2050, elderly people over 80 years old will account for more than 400 million of the world population [28]. One of the challenges for the future is to assure that, despite such a demographic change, mankind can address the health and nutrition security issues posed by an ageing population in a targeted manner. Personalized dietary programs for seniors and technological advances to produce nutritious, palatable, innovative, and affordable food products tailored to the special needs of seniors are among the expected progresses of healthcare systems and food industries for the upcoming years.

In this context, we propose an innovative application of rapeseed proteins that adds new perspectives for the valorization of currently underutilized sustainable protein sources. RSM protein isolate obtained through sustainable and green processes was applied as a supplement for texture-modified (TM) food suitable for elderly people with mastication and swallowing difficulties. The compositional, nutritional, and sensory evaluations of the food products formulated are hereby described. This is a first-of-its-kind application of rapeseed proteins, coupling green and sustainable technologies for the recovery of proteins from agri-food by-products with advanced food formulations delivering personalized nutrition solutions for an ageing population.

## 2. Materials and Methods

### 2.1. RSM Protein Isolate

RSM protein isolate was obtained from the extraction and purification of RSM, a by-product of an oil-pressing factory (Poľnoservis a.s., Leopoldov, Slovakia) serving an adjacent biofuel biorefinery plant. RSM met the legal requirements of the Slovak Government Regulation no. 438/2006, Act 271/2005 Coll., the European Parliament and Council Regulation (EC) no. 767/2009, and the Commission Regulation no. 68/2013 of the Fodders Catalogue.

The original feedstock was a nongenetically modified rapeseed (*Brassica napus* L. var. Napus) with low erucic acid and low glucosinolate contents grown during the 2020/2021 crop season in Central and East European regions, namely Slovakia, Poland, Hungary, Czech Republic, Romania, and Ukraine. Seed quality met the STN 462300-1 and 2 and the Codex Alimentarius requirement of the Slovak Republic, Government Regulation no. 439/2006, and the requirements set out in the list of permitted varieties.

The protein extraction and purification from RSM was performed at the semi-pilot scale on 2–5 kg pre-batch at Celabor (Herve, Belgium). The RSM was first ground using a MASUKO^®^ supercolloider (Masuko Sangyo Co., Ltd., Kawaguchi, Japan). The ground material was extracted using an aqueous alkaline solution in mild conditions, in a 65 L maceration tank (Ferrari srl, Ghislenghien, Belgium). The solid–liquid separation was performed with a vertical centrifuge (RC30, Rousselet-Robatel, Annonay, France). An extract with a yield of 19.0 ± 3.5% was obtained, containing 23.3 ± 4.5% of proteins, determined with the Kjeldahl method. Protein concentrate was obtained with a purity of 76.0 ± 5.1% with isoelectric precipitation, and the following purification step was performed using membrane microfiltration, using 10 L pilot equipment (Evonik Industries AG, Essen, Germany) with a ceramic membrane of 50 kDa. The obtained protein isolate was freeze-dried before analysis with a final yield of 7.2 ± 0.7% and a high purity (batch 1, Figure S1).

**Figure 1:**
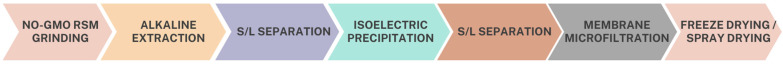
Flow-chart of RSM protein production process.

After validation at the semi-pilot scale, the process was up-scaled on a 50–100 kg full-pilot-scale batch at ENVIRAL a.s. (Leopoldov, Slovak Republic), delivering the final rapeseed meal protein isolate product by means of spray drying (batch 2, Figure S2). The main steps of the RSM protein production process are detailed in Figure 1.

The compositional data of the RSM protein isolates from semi-pilot- and full-pilot-scale trials are reported in Table 1.

The two protein isolates were used for food product formulation by Biozoon GmbH (Bremerhaven, Germany) as described below.

### 2.2. Formulation and Preparation of Texture-Modified Food

Texture-modified (TM) food was formulated and prepared at Biozoon GmbH according to internally established protocols, as described below.

In order to evaluate the suitability of RSM protein isolate as a supplement to TM products, different types of food were preliminary tested. Chicken breast, carrot, and bread were selected for this study. Food matrices were pureed, texture-modified, and tested for their sensory quality and nutritional value without and with supplementation with RSM protein isolate from semi-pilot- and full-pilot-scale tests. All ingredients, except texturizers and RSM protein isolate, were purchased from a local supermarket in Bremerhaven, Germany.

**Figure 2:**
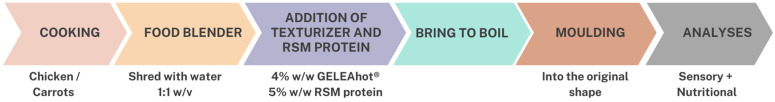
Flow-chart of TM chicken breast and TM carrot preparation.

Steam-cooked and spiced chicken breast and carrots were chopped, added with water (1:1 *w/v*), and pureed in a food blender (Blixer^®^ 3, Robot Coupe, France). RSM protein isolate (5% *w/w*) and GELEAhot instant^®^ (4% *w/w*), a texturizing system owned by Biozoon, were added to the pureed food and homogenized manually with a whisk. GELEAhot instant^®^ is composed of maltodextrin, agar-agar, and xanthan, requiring an activation temperature of approximately 87 °C before forming a gel while cooling. The pureed food with added RSM protein isolate and GELEAhot instant^®^ was brought to boil and then molded into silicone molds resembling the shape of the original food in order to increase the appeal and sensory acceptability of the product. The main preparation steps of the TM chicken breast and carrots are detailed in Figure 2.

The extent of RSM protein supplementation (5% *w/w*) was the one adopted in Biozoon’s internal protocols based on previous experience, indicating this ratio as the one with the best performance, delivering an additional amount of useful protein without significantly affecting the technological and sensory characteristics of the products. The control samples were prepared following the same procedures described above, except the addition of RSM protein.

RSM protein isolate was also tested as a supplement for TM bread, prepared by using Biozoon’s SMOOTHBROT^®^ mix, based on gluten, maltodextrin, whey protein, oil powder, agar-agar, and xanthan gum.

**Figure 3:**
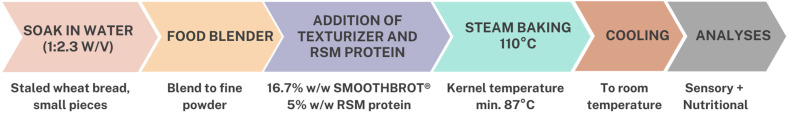
Flow-chart of TM bread preparation.

TM bread (1.2 kg loaf) was prepared as follows. Stale wheat bread, roughly chopped into small pieces, was added to tap water (about 1:2.3 *w/v*) and left to soak for approx. 20 min in a flat bowl. The content of the bowl was then transferred into a blender (Blixer^®^ 3, Robot Coupe, France), mixed to a smooth dough-like mass, added to SMOOTHBROT^®^ mix powder (16.7% *w/w*) and RSM protein isolate (5% *w/w*), and gently stirred manually. The mixture was further transferred into a baking pan and steamed for about 90 min until a kernel temperature of nearly 90 °C was reached. Afterwards, the bread was cooled down to room temperature while resting in the pan to allow the gel structure to form while cooling. The main steps of TM bread preparation are detailed in Figure 3.

### 2.3. Sensory Evaluations

A descriptive sensory analysis was conducted at Biozoon’s laboratories according to the DIN 10964:2014-11 standard method [29]. The evaluations were performed by a group of 5 staff experts trained in sensory analyses. The evaluation group analyzed all three RSM-protein-enriched products in order to identify individual product aspects in terms of descriptive attributes (appearance, odor, taste, and mouthfeel/texture). The attributes were collected correspondingly for further interpretation. Control samples of each product (meaning without any RSM protein supplementation) were presented to the group as well, in order to describe possible differences between the two products.

For the sensory evaluation, RSM-protein-enriched TM chicken breast and TM carrots were reshaped in gel blocks and served as such. TM bread was cut into slices of approx. 0.8 cm (the thickness of a standard bread slice). Each participant in the sensory evaluation was allowed to take as much as needed in order to describe the products. The evaluation group was informed about the purpose of the sensory analyses. Each sample was served separately, and the selection of attributes was free and unbounded to a list. A list of product-specific attributes was further developed in order to identify relevant differences between the supplemented products and their respective controls.

### 2.4. Chemical Analyses

The freshly prepared TM food samples (chicken breast, carrots, and bread) were packed under vacuum and shipped in a refrigerated state to CREA laboratories in Rome (Italy). Upon arrival, the food was immediately frozen and freeze-dried (Scanvac Coolsafe 55-4 Pro, Labogene, Allerød, Denmark) for further chemical analyses. The results were further normalized and expressed on a wet mass basis. The semi-pilot-scale and full-pilot-scale batches of RSM protein isolate were analyzed as received.

The moisture, crude protein, crude fat, and ash contents were determined separately in individual RSM protein isolate batches and in the formulated foods with and without supplementation with RSM protein following the methods of the Association of Official Analytical Chemists [30]. The crude protein content was evaluated using the Kjeldahl procedure, using 6.25 as a nitrogen-to-protein conversion factor. Nonprotein nitrogen (NPN) was determined using the Kjeldahl method after protein precipitation with 10% (*w*/*v*) trichloroacetic acid and filtration. The crude fat content was determined using Soxhlet extraction. The ash content was determined gravimetrically after incineration in a muffle furnace at 550 °C. Total dietary fiber was determined according to the method of Prosky et al. [31]. Carbohydrates were calculated by difference. All macronutrients’ analyses were performed in triplicate. The energy content was calculated by using the conversion factors indicated by the EU Regulation 1169/2011 [32]. The conversion factor from kcal to kJ was 4.184.

Macrominerals and trace elements were quantified using inductively coupled plasma optical emission spectrometry (Optima 8000™ ICP-OES, Perkin-Elmer, Waltham, MA, USA) after liquid ashing in a microwave digestion system (1200 Mega, Milestone srl, Sorisole (BG), Italy). Mineral analyses were performed in quadruplicate.

### 2.5. Quality Assurance

For the validation of the applied methods and quality control of the proximate and dietary fiber data, the standard reference materials peanut butter (NIST 2387, National Institute of Standards and Technology, Gaithersburg, MD, USA) and dried haricot beans (BC514, European Reference Material ERM^®^, Geel, Belgium) were analyzed. For the validation of the method and the quality control of minerals and trace element data, three Certified Reference Materials, cabbage (IAEA-359, International Atomic Energy Agency Reference Materials Group, Vienna, Austria), peanut butter (NIST 2387), and haricots verts (BCR 383, Community Bureau of Reference, Brussels, Belgium), were analyzed. All analyses were performed at least in triplicate.

## 3. Results and Discussion

### 3.1. Food Formulation

The TM chicken breast, carrots, and bread were formulated without and with supplementation with the RSM protein isolate. The formulations of TM chicken breast, carrots, and bread are reported in Table 2, Table 3 and Table 4.

**Figure 4:**
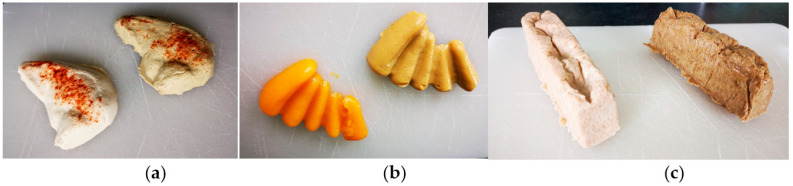
Texture-modified food products without (**left**) and with (**right**) the addition of RSM: (**a**) chicken breast; (**b**) carrots; (**c**) bread.

The TM food without and with RSM protein supplementation (Figure 4) underwent a sensory evaluation at first and then chemical and nutritional evaluations.

### 3.2. Sensory Assessment

The RSM-protein-supplemented TM chicken breast samples were darker in color compared to the control and slightly brownish. No off smell was reported, so the product kept the original smell of chicken. The collected taste attributes of the samples were described as slightly bitter and off-tastes also occurred (strawy), but, still, the product was reported as acceptable with a recognizable original taste. The supplemented TM chicken breast samples were described as softer, with the results being acceptable within TM food applications.

Rapeseed protein supplementation (2%) in beef and pork sausages has been also reported in the literature [22,33] with acceptable results regarding product quality maintenance. In the case of TM carrots, supplementation with 5% RSM protein isolate showed limited applicability, especially with regard to odor and taste. Besides the darker color, which was expected, an off smell as well as seedy notes were reported when the supplementation with RSM protein isolate was used. The characteristic carrot flavor was also masked by the RSM protein addition. These results suggest that, in the case of carrots, an adjustment to the formulation, consisting of a lower supplementation with RSM protein, is advisable.

The texture was described as softer than the control, but still with good uses for texture-modified purposes. To the best of our knowledge, studies on the incorporation of rapeseed protein into vegetable matrices have not been reported in the literature; thus, a comparison on this matter was not possible. As already reported for chicken and carrots, RSM protein supplementation in TM bread also resulted in a darker color compared to the control sample, which was, in this special case, recognized positively due to associations with whole-grain bread.

Similar results were also described by Korus et al. [34], who incorporated different amounts of rapeseed protein (6–15%) as a starch replacer in gluten-free breads and registered improved color characteristics. Furthermore, our study showed that the attribute “whole-grain characteristics” was additionally mentioned in the odor and taste description. The odor was described as bread-like and slightly roasted, which is positive. In terms of the described taste, besides the bread-like characteristics, malty notes and seedy notes were also reported. Nevertheless, the positive attributes with regard to taste must not be disregarded. The reported texture attributes were summarized as slightly drier. This could be due to the increased dry matter of the product resulting from the rapeseed protein supplementation, as described in the next section.

### 3.3. Nutritional Evaluations

The macronutrient composition and energy value of the TM chicken breast, carrots, and bread without and with supplementation with RSM protein are reported in Table 5, Table 6 and Table 7. The results highlight a higher nutrient density in RSM-protein-supplemented food compared to the control samples. In particular, the supplemented products showed higher dry matter (chicken breast: +14–16%; carrots: +45–108%; and bread: +8–9%) and protein (chicken breast: +19–24%; carrots: +1035–1120%; and bread: +38–41%) contents compared to the control samples.

With the addition of the RSM protein isolate, the chicken breast and bread showed a slight increment in the energy value (chicken: +10–15%, depending on the protein batch used; bread: +7–10%) and no relevant changes in the other macronutrients (Table 5 and Table 7). The TM carrots (Table 6) were the product that received more nutritional advantage from supplementation with rapeseed meal protein isolate, not only in terms of protein content (with an over 10-fold increment) but also of total minerals (ashes +72–87%).

Similar trends as regards the macronutrient profile were observed in the two independent trials carried out to test the semi-pilot and full-pilot batches of RSM protein isolates. This is an indication of the robustness and reproducibility of the protein extraction process and the reliability of the formulations used.

The mineral profile of the TM food without and with the addition of RSM protein is reported in Table 8, Table 9 and Table 10.

With the addition of RSM protein isolate, the TM chicken breast showed an increment of minerals (Table 8), in particular phosphorus and copper, to different extents depending on the protein batch used.

The TM carrots added with RSM protein (Table 9) showed a higher content of most minerals and trace elements, in particular phosphorus, zinc, and copper.

The RSM-protein-fortified bread (Table 10) also showed a nutritionally favorable increment in minerals in the two trials, in particular phosphorus and copper, reflecting the peculiar composition of the semi-pilot and full-pilot RSM protein isolate batches.

The higher nutrient density of RSM-protein-supplemented food is a nutritionally favorable attribute, considering that the texture modification of food implies the addition of a high amount of water and that, as a consequence, TM products have a low nutrient density compared to the original food matrix [35].

The increased protein and mineral contents of TM products with added RSM protein isolate reported here are nutritionally correct and adequate since, while a lower energy intake is needed at an advanced age, the micronutrient and protein requirements are not diminished [36]. In fact, an adequate protein intake is necessary at an advanced age to prevent the loss of muscle mass, a frequent negative health concern of ageing [37].

Rapeseed proteins napin and cruciferin, the major storage proteins of rapeseed, have a balanced amino acid composition, and a protein efficiency ratio comparable to that of other proteins commonly used in food preparations such as egg and milk proteins [16]. Therefore, supplementation with rapeseed protein isolate has a positive impact on the nutritional properties of the TM food. In addition, besides providing all essential amino acids, supplementation with RSM protein gives products an added value in functional terms because, once digested, rapeseed proteins are potentially cleaved into bioactive peptides with beneficial health properties such as antihypertensive, antioxidant, bile-acid-binding, and antithrombotic [33,38,39,40].

An adequate intake of dietary fiber is important at any age to increase bowel motility in order to prevent constipation and chronic diseases typical of older people [41]. Thus, targeting adequate protein and fiber intakes is of pivotal importance at an advanced age.

As regards minerals, the contribution given by rapeseed protein isolate added to food is also favorable. In chicken breast and bread, RSM protein supplementation positively affected the content of phosphorus, calcium, and copper, minerals essential in bone mineralization, oxygen transport, energy metabolism, and enzyme activities. Adequate levels of these minerals in the diet have beneficial implications for the elderly. The copper values in the RSM-protein-supplemented samples (about 0.2 mg 100 g^−1^) were largely within the nutritionally recommended levels (corresponding to 0.9 mg/day for adults) and comparable to those present in several foods of animal and plant origin (i.e., contents per 100 g: pork meat, 0.15 mg; beans, 0.7 mg; barley, 0.29 mg; and carrots, 0.19 mg) [42]. The increment in sodium observed in the carrots and bread supplemented with batch 1 of the RSM protein isolate (by 50% and 16%, respectively) does not represent a serious health concern, as the detected levels (200–240 mg per 100 g product), in the frame of a daily diet, are very far from the maximum advisable levels of sodium for hypertension prevention established at 2 g/day.

Meat, vegetable, and bread consumption may be limited in old age because of mastication and swallowing difficulties, especially in patients with dysphagia problems. This may lead to protein, energy, dietary fiber, vitamin, and mineral deficits. Texture modification combined with the rapeseed protein fortification of food has a positive impact on the nutritional profile of the food and increases the palatability, acceptability, and nutrient density of the diet for patients affected by dysphagia or with mastication difficulties. Furthermore, the texture modification steps imply a modification of the initial food matrices from solid to fluidic and, finally, to special texture (e.g., gel), with such a procedure being highly advantageous as the fluidic stage offers the perfect condition for allowing fast, high, and specific ingredient supplementation. These are unique features with potential in future applications of tailor-made food with a high nutritional profile.

The enhanced protein and mineral contents of food reported here are in line with the current dietary recommendations for elderly people [43,44,45]. Clinical studies demonstrate that elderly people are at risk of malnutrition. Physiological changes, a decline in physical activity, and a loss of appetite and taste sensitivity are only some of the factors that expose seniors at an increased risk of nutritional inadequacy in advanced age [46].

Elderly people with mastication and swallowing difficulties are a category at increased risk of malnutrition. The use of pureed food as a nutritional solution is very limited as it is not adequately formulated and does not address the key requirements of a food (e.g., sensorially pleasant). The careful design and formulation of TM food are, therefore, needed in order to give it the desired nutritional and sensory properties [47] as well as provide the motivation to eat it [48]. Furthermore, protein supplementation based on the specific needs of seniors can be applied to counteract the prevalence of malnutrition in this population group. Here, the use of rapeseed protein has potential. Furthermore, malnourished elderly people are most likely not able to consume an entire meal; thus, smaller portions supplemented with key ingredients as proteins are necessary. Moreover, there is a connection between suffering from eating difficulties (e.g., dysphagia) and a decrease in overall food intake, which underlines the need for supplemented texture-modified foods for such groups of elderly people [48,49].

## 4. Conclusions

Food consumption may be limited at old age because of mastication and swallowing difficulties. This may lead to protein, energy, dietary fiber, vitamin, and mineral deficiencies. Texture modification increases the palatability, acceptability, and nutrient density of the diet of aged people affected by dysphagia or with mastication difficulties. Protein supplementation based on the specific needs of seniors can be combined with texture modification in order to design highly nutrient-dense food and counteract the prevalence of malnutrition in this population group.

This study highlights the potentialities of RSM protein isolate as a food supplement for TM food for elderly people with mastication and dysphagia problems.

The obtained results show that the texture modification of food combined with rapeseed protein isolate supplementation may have a positive impact on the nutritional and sensory profile of food. The consistency of the obtained results in terms of protein enrichment of TM food in the two independent trials, testing RSM protein isolates obtained from semi-pilot and full-pilot-scale extractions and purifications, is an indication of the robustness of the processes and the reliability of the formulations.

Within the tested food applications, TM chicken breast and bread were the products giving the best results, showing unaltered or even improved sensory properties and a richer nutritional profile with special regard to the protein and mineral contents. On the contrary, supplemented TM carrots, in spite of the higher nutrient density, showed limited acceptability due to poor sensory properties that could be overcome with an adjustment to the formulation.

The RSM protein isolate applied as an ingredient to TM food in this study is the main output of a biorefining process aimed at recovering and valorizing underutilized nutrients present in an agri-food by-product such as RSM. The application of RSM protein proposed here is in line with the current major societal challenges, such as the responsible management of natural resources, the valorization of agri-food by-products, and healthy nutrition with a focus on elderly people.

## Figures and Tables

**Table 1:** Macronutrient and mineral composition of rapeseed meal (RSM) protein isolate from semi-pilot- and full-pilot-scale trials. Values expressed per 100 g of product.

		RSM Protein Isolate	
	Unit	Batch 1 (Semi-Pilot-Scale)	Batch 2 (Full-Pilot-Scale)
		mean ± SD	mean ± SD
Moisture	g	4.75 ± 0.02	3.35 ± 0.05
Crude protein	g	85.72 ± 0.34	81.72 ± 0.66
Nonprotein N	g	0.59 ± 0.11	0.29 ± 0.08
Ash	g	0.97 ± 0.17	3.22 ± 0.07
Crude fat	g	0.75 ± 0.06	1.69 ± 0.07
Total dietary fiber	g	3.20 ± 0.26	1.70 ± 0.17
Carbohydrate	g	4.61 ± 0.20	8.34 ± 0.57
Potassium	mg	26.88 ± 5.38	18.07 ± 2.92
Phosphorus	mg	743.18 ± 41.17	1370.88 ± 29.28
Sodium	mg	18.22 ± 0.40	3.55 ± 0.19
Magnesium	mg	6.73 ± 0.46	1.72 ± 0.21
Calcium	mg	91.61 ± 1.52	9.23 ± 0.66
Zinc	mg	0.73 ± 0.01	1.29 ± 0.03
Manganese	mg	0.18 ± 0.00	0.07 ± 0.03
Copper	mg	2.85 ± 0.07	1.83 ± 0.05

**Table 2:** Recipe of texture-modified (TM) chicken breast without (control) and with the addition of RSM protein isolate. Values are expressed as g 100 g^−1^ of fresh product.

	TM Chicken Control	TM Chicken + RSM Protein
Chicken breast (g)	47.2	44.7
Water (g)	48.0	45.5
GELEAhot instant^®^ (g)	4.0	4.0
RSM protein (g)	-	5.0
Sunflower oil (g)	0.8	0.8

**Table 3:** Recipe of TM carrots without (control) and with the addition of RSM protein isolate. Values are expressed as g 100 g^−1^ of fresh product.

	TM Carrots Control	TM Carrots + RSM Protein
Carrots (g)	48.0	45.5
Water (g)	48.0	45.5
GELEAhot instant^®^ (g)	4.0	4.0
RSM protein (g)	-	5.0

**Table 4:** Recipe of TM bread without (control) and with the addition of RSM protein isolate. Values are expressed as g 100 g^−1^ of fresh product.

	TM Bread Control	TM Bread + RSM Protein
Bread (g)	25.0	23.5
Water (g)	58.3	54.8
SMOOTHBROT^®^ mix (g)	16.7	16.7
RSM protein (g)	-	5.0

**Table 5:** Macronutrient composition and energy value of TM chicken breast: control and RSM-protein-supplemented (5% *w/w*) samples. Values are expressed per 100 g of fresh product.

	Batch 1 (Semi-Pilot)		Batch 2 (Full Pilot)	
	TM Chicken Control	TM Chicken + RSM Protein	TM Chicken Control	TM Chicken + RSM Protein
	mean ± SD	mean ± SD	mean ± SD	mean ± SD
pH	6.14 ± 0.01	5.89 ± 0.10	6.01 ± 0.15	5.63 ± 0.00
Dry matter (g)	22.95 ± 0.10	26.65 ± 0.01	23.45 ± 0.19	26.64 ± 0.39
Moisture (g)	77.05 ± 0.10	73.35 ± 0.10	76.55 ± 0.19	73.37 ± 0.39
Crude protein (g)	15.73 ± 0.20	19.53 ± 0.28	15.82 ± 0.34	18.80 ± 0.41
Carbohydrates (g)	3.08 ± 0.20	3.09 ± 0.20	1.95 ± 0.10	1.86 ± 0.10
Ash (g)	1.07 ± 0.03	1.10 ± 0.07	1.03 ± 0.04	1.20 ± 0.01
Crude fat (g)	1.87 ± 0.04	1.73 ± 0.02	2.60 ± 0.02	2.40 ± 0.08
Total dietary fiber (g)	1.20 ± 0.04	1.20 ± 0.18	2.05 ± 0.35	2.37 ± 0.37
Energy value:				
kcal	94	108	99	109
kJ	393	452	412	456

**Table 6:** Macronutrient composition and energy value of TM carrots: control and RSM-protein-supplemented (5% *w/w*) samples. Values are expressed per 100 g of fresh product.

	Batch 1 (Semi-Pilot)		Batch 2 (Full Pilot)	
	TM Carrots Control	TM Carrots + RSM Protein	TM Carrots Control	TM Carrots + RSM Protein
	mean ± SD	mean ± SD	mean ± SD	mean ± SD
pH	6.45 ± 0.01	5.44 ± 0.01	6.73 ± 0.02	4.74 ± 0.04
Dry matter (g)	8.40 ± 0.06	17.45 ± 0.10	9.16 ± 0.10	13.32 ± 0.02
Moisture (g)	91.60 ± 0.06	82.55 ± 0.12	90.84 ± 0.10	86.68 ± 0.02
Crude protein (g)	0.46 ± 0.08	5.62 ± 0.08	0.37 ± 0.04	4.20 ± 0.06
Carbohydrates (g)	5.18 ± 0.20	7.47 ± 0.25	5.69 ± 0.15	5.81 ± 0.20
Ash (g)	0.65 ± 0.07	1.12 ± 0.01	0.23 ± 0.02	0.43 ± 0.01
Crude fat (g)	0.10 ± 0.01	0.10 ± 0.01	0.10 ± 0.02	0.10 ± 0.02
Total dietary fiber (g)	2.01 ± 0.02	3.14 ± 0.14	2.77 ± 0.11	2.78 ± 0.28
Energy value:				
kcal	27	60	31	47
kJ	113	251	128	195

**Table 7:** Macronutrient composition and energy value of TM bread: control and RSM-protein-supplemented (5% *w/w*) samples. Values are expressed per 100 g of fresh product.

	Batch 1 (Semi-Pilot)		Batch 2 (Full Pilot)	
	TM Bread Control	TM Bread + RSM Protein	TM Bread Control	TM Bread + RSM Protein
	mean ± SD	mean ± SD	mean ± SD	mean ± SD
pH	5.87 ± 0.01	5.53 ± 0.02	5.74 ± 0.21	4.80 ± 0.06
Dry matter (g)	38.02 ± 0.10	41.41 ± 0.10	33.28 ± 0.12	36.03 ± 0.72
Moisture (g)	61.98 ± 0.21	58.59 ± 0.30	66.72 ± 0.12	63.97 ± 0.72
Crude protein (g)	10.78 ± 0.15	14.90 ± 0.29	8.17 ± 0.13	11.51 ± 0.11
Carbohydrates (g)	18.36 ± 0.16	17.79 ± 0.05	17.79 ± 0.15	16.94 ± 0.10
Ash (g)	0.94 ± 0.16	0.88 ± 0.01	0.65 ± 0.04	0.72 ± 0.02
Crude fat (g)	3.11 ± 0.10	3.09 ± 0.01	2.31 ± 0.02	2.19 ± 0.04
Total dietary fiber (g)	4.83 ± 0.21	4.75 ± 0.26	4.36 ± 0.06	4.67 ± 0.05
Energy value:				
kcal	154	168	133	143
kJ	644	703	558	598

**Table 8:** Mineral and trace element contents of TM chicken breast: control and RSM-protein-supplemented (5% *w/w*) samples. Values are expressed as mg per 100 g of fresh product.

	Batch 1 (Semi-Pilot)		Batch 2 (Full Pilot)	
	TM Chicken Control	TM Chicken + RSM Protein	TM Chicken Control	TM Chicken + RSM Protein
	mean ± SD	mean ± SD	mean ± SD	mean ± SD
Potassium	295.02 ± 1.96	283.44 ± 3.44	278.22 ± 11.64	248.48 ± 1.97
Phosphorus	159.23 ± 2.40	192.95 ± 11.64	144.88 ± 5.03	205.68 ± 2.42
Sodium	171.51 ± 0.87	167.60 ± 0.23	119.13 ± 3.03	107.23 ± 0.72
Magnesium	23.92 ± 0.09	23.57 ± 0.01	21.82 ± 0.35	19.80 ± 0.22
Calcium	22.22 ± 0.44	27.18 ± 0.40	18.14 ± 0.48	17.10 ± 0.03
Zinc	0.39 ± 0.02	0.41 ± 0.00	0.40 ± 0.01	0.43 ± 0.00
Manganese	0.03 ± 0.00	0.04 ± 0.00	0.03 ± 0.00	0.03 ± 0.00
Copper	0.02 ± 0.00	0.19 ± 0.01	0.02 ± 0.00	0.11 ± 0.00

**Table 9:** Mineral and trace element contents of TM carrots: control and RSM-protein-supplemented (5% *w/w*) samples. Values are expressed as mg per 100 g of fresh product.

	Batch 1 (Semi-Pilot)		Batch 2 (Full Pilot)	
	TM Carrots Control	TM Carrots + RSM Protein	TM Carrots Control	TM Carrots + RSM Protein
	mean ± SD	mean ± SD	mean ± SD	mean ± SD
Potassium	163.87 ± 8.82	242.09 ± 9.40	63.54 ± 1.31	57.75 ± 2.36
Phosphorus	14.01 ± 0.93	73.55 ± 0.29	8.73 ± 0.17	79.33 ± 9.71
Sodium	158.00 ± 12.12	240.71 ± 8.74	23.65 ± 0.54	21.91 ± 0.47
Magnesium	5.68 ± 0.33	8.23 ± 0.12	4.89 ± 0.05	4.45 ± 0.06
Calcium	30.11 ± 2.70	50.12 ± 0.34	30.22 ± 0.16	27.28 ± 0.13
Zinc	0.12 ± 0.01	0.22 ± 0.01	0.11 ± 0.00	0.31 ± 0.01
Manganese	0.08 ± 0.01	0.13 ± 0.00	0.07 ± 0.00	0.07 ± 0.00
Copper	0.01 ± 0.00	0.23 ± 0.00	0.01 ± 0.00	0.21 ± 0.00

**Table 10:** Mineral and trace element contents of TM bread: control and RSM-protein-supplemented (5% *w/w*) samples. Values are expressed as mg per 100 g of fresh product.

	Batch 1 (Semi-Pilot)		Batch 2 (Full Pilot)	
	TM Bread Control	TM Bread + RSM Protein	TM Bread Control	TM Bread + RSM Protein
	mean ± SD	mean ± SD	mean ± SD	mean ± SD
Potassium	125.17 ± 18.45	127.12 ± 6.61	107.79 ± 0.46	100.85 ± 2.34
Phosphorus	60.10 ± 8.32	105.57 ± 3.17	47.05 ± 0.22	107.74 ± 3.09
Sodium	175.35 ± 4.56	203.69 ± 5.23	124.14 ± 1.41	118.87 ± 2.69
Magnesium	22.99 ± 1.40	20.82 ± 0.48	14.90 ± 0.31	14.50 ± 0.13
Calcium	68.55 ± 1.13	72.52 ± 1.56	43.61 ± 0.36	43.87 ± 0.96
Zinc	0.68 ± 0.07	0.72 ± 0.01	0.48 ± 0.01	0.73 ± 0.03
Manganese	0.65 ± 0.10	0.68 ± 0.02	0.43 ± 0.01	0.43 ± 0.00
Copper	0.07 ± 0.00	0.24 ± 0.01	0.08 ± 0.00	0.22 ± 0.01

## Data Availability

The data presented in this study are available in the article and in the Supplementary Materials.

## Supplementary Materials

The following supporting information can be downloaded at: https://www.mdpi.com/article/10.3390/foods12061326/s1, Figure S1: Rapeseed meal protein isolate from semi-pilot plant used in this study as an ingredient of texture-modified food; Figure S2: Rapeseed meal protein isolate from full-pilot plant used in this study as an ingredient of texture-modified food.
