# Supplementary material for: Application of Rapeseed Meal Protein Isolate as a Supplement to Texture-Modified Food for the Elderly
Source: Foods. 2023 Mar 20;12(6):1326. doi: 10.3390/foods12061326 (PMC10048395; doi:10.3390/foods12061326)
Supplement: Supplementary file 1 [file foods-12-01326-s001.zip › foods-2241076-Supplementary.pdf]

Supplementary Information

Application of Rapeseed Meal Protein Isolate as a Supplement to Texture-Modified Food for the Elderly

Gabriella Di Lena [1,*], Ann-Kristin Schwarze [2], Massimo Lucarini [1], Paolo Gabrielli [1], Altero Aguzzi [1], Roberto Caproni [1], Irene Casini [1], Stefano Ferrari Nicoli [1], Darleen Genuttis [2], Petra Ondrejíčková [3], Mahmoud Hamzaoui [4], Camille Malterre [4], Valentína Kafková [5] and Alexandru Rusu [2]

[1] CREA Research Centre for Food and Nutrition, Via Ardeatina 546, 00178 Rome, Italy; massimo.lucarini@crea.gov.it (M.L.); paolo.gabrielli@crea.gov.it (P.G.); altero.aguzzi@crea.gov.it (A.A.); roberto.caproni@crea.gov.it (R.C.); irene.casini@crea.gov.it (I.C.); stefano.nicoli@crea.gov.it (S.F.N.)
[2] Biozoon GmbH, Nansenstraße 8, 27572 Bremerhaven, Germany; schwarze@biozoon.de (A.-K.S.); genuttis@biozoon.de (D.G.); rusu@biozoon.de (A.R.)
[3] ENVIRAL a.s., Trnavská cesta, 920 41 Leopoldov, Slovakia; ondrejickova@enviengroup.eu
[4] Celabor, Avenue du Parc 38, 4650 Herve, Belgium; mahmoud.hamzaoui@celabor.be (M.H.); camille.malterre@celabor.be (C.M.)
[5] Centrum výskumu a vývoja, s. r.o. (Centre for Research and Development), Trnavská Cesta 1033/7, 920 41 Leopoldov, Slovakia; kafkova@enviengroup.eu
* Correspondence: gabriella.dilena@crea.gov.it; Tel.: +39-06-51494501

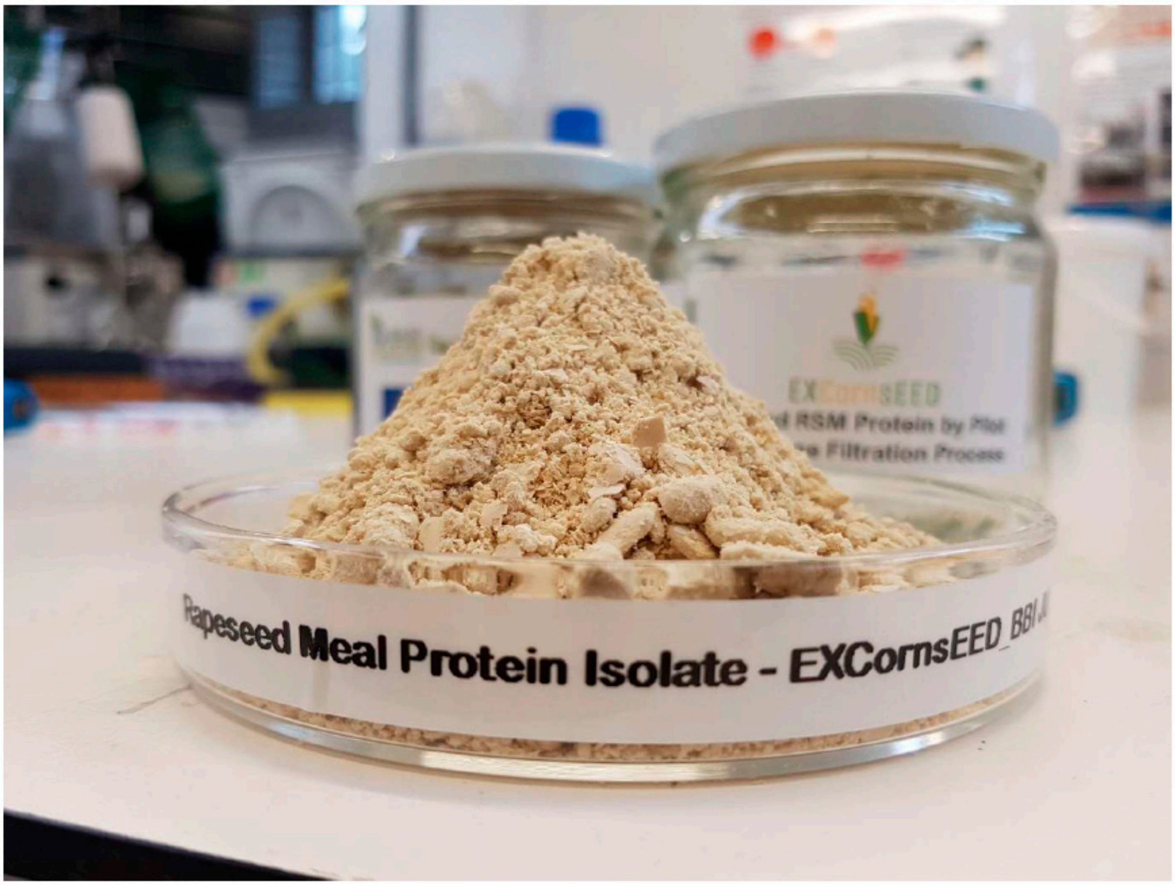

Figure S1. Rapeseed meal protein isolate from semi-pilot plant used in this study as an ingredient of texture-modified food.

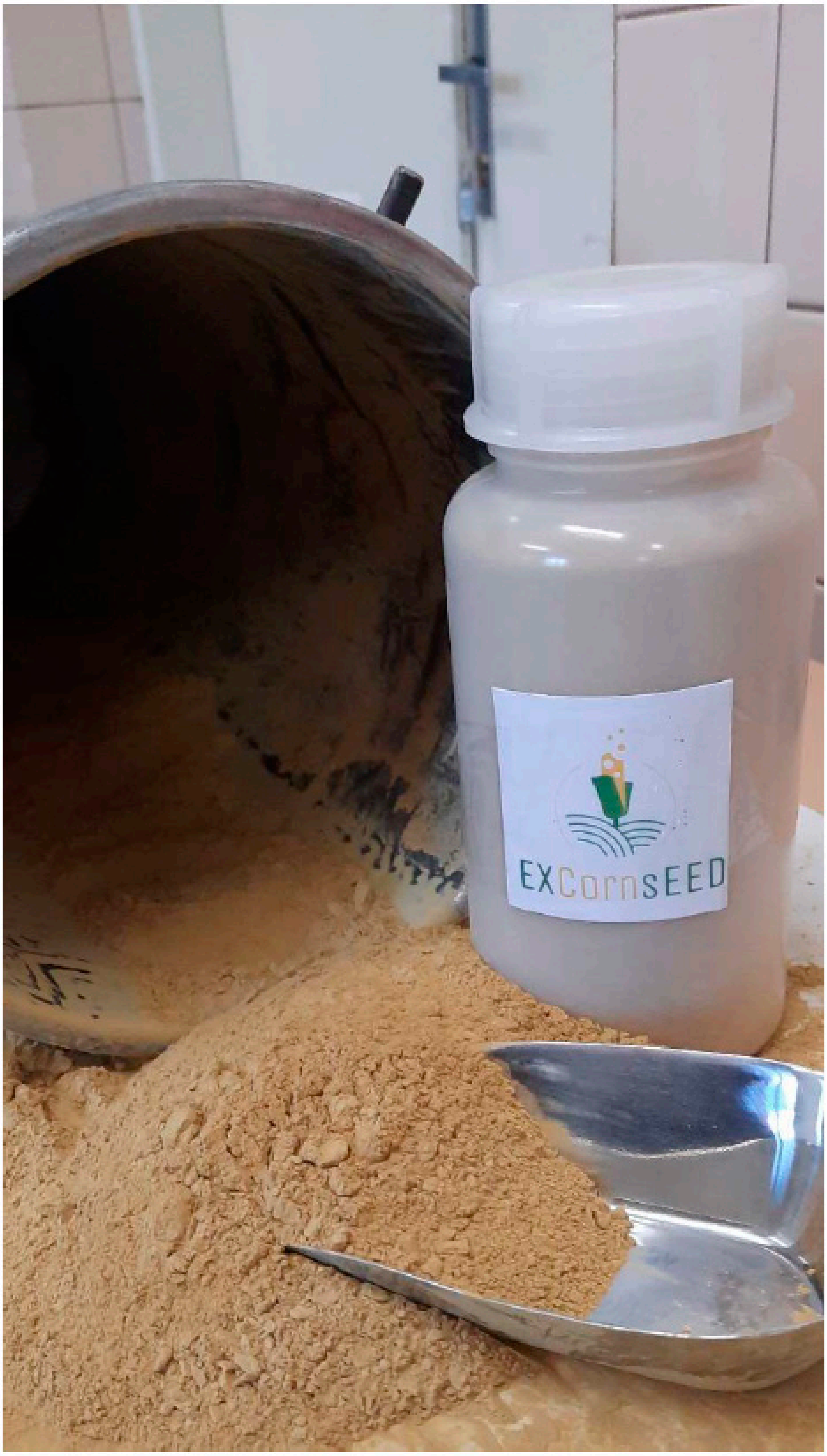

Figure S2. Rapeseed meal protein isolate from full-pilot plant used in this study as an ingredient of texture-modified food.
